# Recent advances in using diffusion tensor imaging to study white matter alterations in Parkinson’s disease: A mini review

**DOI:** 10.3389/fnagi.2022.1018017

**Published:** 2023-02-22

**Authors:** Yao-Chia Shih, Wen-Yih Isaac Tseng, Leila Montaser-Kouhsari

**Affiliations:** ^1^Graduate Institute of Medicine, Yuan Ze University, Taoyuan, Taiwan; ^2^AcroViz Inc., Taipei, Taiwan; ^3^Institute of Medical Device and Imaging, National Taiwan University College of Medicine, Taipei, Taiwan; ^4^Department of Neurology, Stanford University, Palo Alto, CA, United States

**Keywords:** Parkinson’s disease, diffusion tenor imaging, structural imaging, diffusion MRI tractography, Parkinson’s disease subtypes, Parkinson’s disease stages

## Abstract

Parkinson’s disease (PD) is the second most common age-related neurodegenerative disease with cardinal motor symptoms. In addition to motor symptoms, PD is a heterogeneous disease accompanied by many non-motor symptoms that dominate the clinical manifestations in different stages or subtypes of PD, such as cognitive impairments. The heterogeneity of PD suggests widespread brain structural changes, and axonal involvement appears to be critical to the pathophysiology of PD. As α-synuclein pathology has been suggested to cause axonal changes followed by neuronal degeneration, diffusion tensor imaging (DTI) as an *in vivo* imaging technique emerges to characterize early detectable white matter changes due to PD. Here, we reviewed the past 5-year literature to show how DTI has helped identify axonal abnormalities at different PD stages or in different PD subtypes and atypical parkinsonism. We also showed the recent clinical utilities of DTI tractography in interventional treatments such as deep brain stimulation (DBS). Mounting evidence supported by multisite DTI data suggests that DTI along with the advanced analytic methods, can delineate dynamic pathophysiological processes from the early to late PD stages and differentiate distinct structural networks affected in PD and other parkinsonism syndromes. It indicates that DTI, along with recent advanced analytic methods, can assist future interventional studies in optimizing treatments for PD patients with different clinical conditions and risk profiles.

## Introduction

Parkinson’s disease (PD) is the second most common age-related neurodegenerative disease and has no cure. In addition to the cardinal motor manifestations of PD, non-motor symptoms involving cognitive impairment, autonomic dysfunction, and sleep disorders come to dominate the clinical manifestations, with the progression of neurodegeneration and advancing disease (Williams-Gray et al., 2007; Kalia and Lang, 2015). The abnormal α-synuclein aggregation has been found in the brains of patients with PD in post-mortem autopsies of the brain. Although detecting these aggregates in a live human brain using imaging has been challenging, other imaging techniques, such as diffusion-weighted magnetic resonance imaging (DWI) have advanced, allowing *in vivo* characterization of microstructural changes (Le Bihan et al., 2001) caused by PD, such as axonal involvement (Chung et al., 2009). Axonal involvement appears to be important in the pathophysiology of PD and manifests as white matter changes (Nigro et al., 2016; Tagliaferro and Burke, 2016; Lanskey et al., 2018; Koirala et al., 2019; Zhang and Burock, 2020). Moreover, a plausible PD mechanism known as retrograde axonal degeneration (Tagliaferro and Burke, 2016) has been suggested that α-synuclein accumulation may begin in the presynaptic terminals, causing axonal transporter changes and following axonal degeneration before affecting neurons (Chung et al., 2009; Nigro et al., 2016). Therefore, white matter changes could be measured as an early detectable structural change in PD using the DWI technique (Burke and O'malley, 2013; Lanskey et al., 2018).

Diffusion-weighted magnetic resonance imaging is sensitive to the random motion of water molecules in tissues, allowing a quantitative means to describe tissue microstructural characteristics (Le Bihan et al., 2001). Diffusion tensor imaging (DTI) is the most common method using a single-tensor model to measure diffusion tensors from DWI data and provides four different typical quantitative DTI metrics as follows (Basser and Pierpaoli, 1996): fractional anisotropy (FA) describes the orientation distribution of organic tissues; mean diffusivity (MD) represents the general water diffusion of tissues that can reflect the degree of axonal/neuronal loss; axial diffusivity (AD) and radial diffusivity (RD) respectively describe the diffusion of water molecules along the principle and transverse axis. Furthermore, diffusion MRI tractography (DT) enables noninvasive visualization of *in-vivo* white matter bundle connections (Basser et al., 2000). Based on DWI data, it has been widely applied to facilitate the detection of axonal abnormalities along the tract bundle and assist in guiding interventional treatments for PD patients [e.g., deep brain stimulation (DBS; Calabrese, 2016)].

In this review, we attempt to provide a comprehensive overview of how different DTI analytic methods and tractography have recently been used in (1) detecting axonal deterioration in different stages and subtypes of PD, (2) differentiating patients with idiopathic PD or other parkinsonism disorders, and (3) improving interventional treatments.

## Materials and methods

We performed a systematic search in the PubMed database, with the following key terms: “Parkinson’s Disease” and “diffusion MRI”; or “Parkinson’s Disease” and “diffusion tensor”; or “Parkinson’s Disease” and “diffusion tractography.” Since this is a narrative review, objective-systematic reviews/meta-analysis methods to extract articles do not apply. Thus, the articles were thereby hand-selected when meeting the following criteria: studies published after 2018, published in the English language, article types of original research and randomized clinical trial, topic focusing on white matter microstructures, and studies using typically clinical DTI acquisition. The selected articles with regard to DTI changes in different PD conditions are listed in Table 1.

**Table 1:** Summary of diffusion tensor imaging studies in PD with different clinical conditions.

Studies	Patient group	Age (years)	Disease duration (years or months)	UPDRS-III	H&Y	DTI analytic method	Number of diffusion directions	*b*-value (the degree of diffusion weighting, s/mm^2^)	Main result
DTI changes in different PD stages									
Isaacs et al. (2019)	70 PD	62.01 (8.62)	6.51 (4.64) years	[ON] 14.46 (7.03), [OFF] 29.84 (12.18)	N.A.	Probabilistic tractography, TBA	60	0/1000	- [PD vs. HC] lower FA in STN-IFG, STN-ACC, STN-DLPFC, STN-preSMA in PD; no significant difference in tract strength
Ji et al. (2019)	57 PD	59.50 (1.21)	4.60 (0.61) years	25.00 (1.41)	[mean (SD)] 1.30 (0.35)	TBSS, graph theory	60	0/1000	-[PD vs. HC] lower structural-functional coupling in left CST, higher small-worldness in PD
Scamarcia et al. (2022)	154 PD	61.58 (7.95)	4.95 (4.84) years	28.59 (15.81)	[mean (SD)] baseline: 1.70 (0.81)	TBSS	N.A.	N.A.	-[PD vs. HC, baseline] Lower FA and higher diffusivity metrics in both WMH and NAWM areas in PD. - No significant progressive changes in all DTI metrics after 1–4 years
Shang et al. (2021)	25 PD	65.96 (14.77)	17.44 (5.19) months	26.88 (8.73)	[mean (SD)] 1.28 (0.45)	Deterministic tractography, TBA	32	0/1000	-[PD vs. HC] lower FA in the nigrostriatal projection associated with lower striatal standardized uptake value ratio in PD
Xiao et al. (2021)	PPMI early drug-naïve PD data: 141 drug-naïve PD	61.70 (8.90)	N.A.	20.80 (9.00)	[mean (SD)] 1.60 (0.50)	FBA for SST-CSD, VBA for DTI	64	0/1000	-[PD vs. HC] higher FC and FDC in PLIC, cingulum, CST, SCP in the contralateral side; higher FDC in the ALIC in the affected side; lower FDC in the contralateral cingulum - [L-PD vs. R-PD] higher FD and FDC in CST, SCP, and cingulum in the affected side in R-PD; higher FA and lower MD in the affected side in R-PD
**DTI changes in different motor phenotypes**									
Nazmuddin et al. (2021)	54 PIGD-PD, 44 TD-PD	66.90 (9.26), 62.70 (8.28)	N.A.	32.09 (11.17), 31.32 (12.52)	[subject number, stage 1/2/3/4/5] 15/60/19/6/0, 39/59/0/2/0	VBA, TBA	30	0/1000	-[PIGD-PD vs. TD-PD] lower FA in the bilateral proximal-medial and-lateral NBM-WM tract, no significant MD difference - Low FA and high MD in the NBM-WM tract correlated with severe postural and gait symptoms in PIGD-PD - Significant relationships between NBM-WM integrity, attentional function, and gait impairment in *de novo* PD
Wen et al. (2018)	PPMI early drug-naïve PD data: 52 TD-PD, 13 PIGD-PD	60.46 (9.57), 66.66 (10.17)	7.52 (8.00), 6.54 (6.78) months	19.77 (9.48), 22.46 (8.83)	[subject number, stage 1/2] 26/26, 4/9	TBSS, graph theory, network-based analysis	64	0/1000	-[TD-PD vs. HC or PIGD-PD] regional FA increases and AD/RD decreases in TD-PD - [PIGD-PD vs. HC or TD-PD] widespread FA decreased and AD/RD increases in PIGD-PD - [PIGD-PD vs. TD-PD] PIGD-PD showed more impaired WM tracts, with stronger correlations with UPDRS-III
Yang et al. (2021)	34 PD-NC,19 TD-NC, 15 PIGD-NC	57.50 (7.79), 57.26 (7.82), 57.80 (8.03)	12.79 (6.29), 12.53 (5.63), 13.13 (7.22)	[ON] 33.23 (12.81), 33.57 (12.21), 32.38 (13.42) - [OFF] 39.71 (12.64), 40.68 (13.71), 38.47 (11.47)	N.A.	ROI analysis, probability tractography	33	0/1000	-Altered WM structures in the executive network in PD - [PIGD vs. TD] higher MD and RD in the executive network WM connections in PIGD - A positive correlation between MD in the SLF and verbal fluency task scores in PD-NC
**DTI changes relevant to non-motor symptoms**									
Bledsoe et al. (2018)	23 PD-NC, 35 PD-MCI, 17 PD-D	72.90 (5.80), 74.50 (6.10), 74.50 (6.70)	9.70 (4.40), 9.40 (4.10), 13.50 (4.90) years	26.50 (8.70), 35.30 (7.60), 44.00 (14.60)	[range] 2–3, 2–4, 2–5	ROI analysis	26	0/800	-[PD-NC vs. HC] no significant difference - [PD-MCI or PD-D vs. HC] higher AD and RD in the most anterior segments of CC in PD-MCI and PD-D - [PD-D vs. HC] higher AD and RD in the anterior 2 segments in PD-D - [All PD] DTI changes in the most anterior and posterior segments in associations with cognitive deficits
Chondrogiorgi et al. (2019)	40 PD-NC, 21 PD-D	68.40 (6.00), 70.90 (5.70)	5.70 (4.80), 7.90 (6.80) years	N.A.	[mean] 2.2, 2.5	TBSS	16	0/700	-[PD-D vs. PD-NC] lower FA in CC, CR, and cingulum in PD-D - [All PD] lower cognitive performance in relation to FA/MD/AD changes in CC, prefrontal and limbic WM
Holtbernd et al. (2021)	30 RBD, 29 PD (from multiple sites)	66.80 (9.10), 63.50 (8.30)	146.90 (121.40), 89.90 (85.70) months	2.30 (1.80), 21.30 (9.70)	N.A., 1.80 (0.70)	ROI analysis	[site 1/2/3/4/5/6] = 60/120/54/12030/64	0/1000	-[RBD vs. HC or PD] higher FA in the bilateral ICP, MCP, SCP in RBD - [RBD vs. PD] higher FA in the bilateral SCP and right ICP in RBD - [RBD or PD vs. HC] lower FA in CC and higher FA in right CST in both patient groups
Inguanzo et al. (2021)	15 PD1, 21 PD2, 26 PD3 (data-driven subtypes, based on whole brain analysis maps)	[mean (median)] 75.00 (14.00), 68.00 (9.00), 58.50 (11.00)	[mean (median)] 7.00 (7.50), 9.00 (9.00), 7.00 (5.50) years	[mean (median)] 30.00 (1.00), 29.00 (2.00), 30.00 (2.00)	[subject number, stage 1/2/2.5/3] 1/6/1/4, 1/10/0/9, 6/14/0/6	TBSS	30	0/1000	-[PD1 vs. HC] PD1 with the worst cognition accompanying with widespread FA reductions in the fronto-occipital WM tracts. - [PD2 or PD3 vs. HC] no significant FA changes - [% of PD-MCI] 67, 48, 31%
Minett et al. (2018)	93 PD-NC, 27 PD-MCI	64.30 (10.80), 70.50 (8.10)	18.20 (1.30), 19.00 (1.50) months	25.90 (1.10), 29.20 (2.20)	1.90 (0.10), 2.30 (0.10)	TBSS	64	0/1000	-[All PD vs. HC] higher MD in bilateral CR, IC, EC, CC, IFOF, SFOF, FM, cingulum, SLF, ILF - [PD-MCI vs. PD-NC] no significant difference at baseline, but PD-MCI showing significant MD increases in frontal WM after 18 months - [PD-MCI vs. HC] lower FA in aforementioned tracts as well as CST - Baseline MD is a neural correlate of cognitive function and a predictor of motor decline
Ohlhauser et al. (2019)	PPMI data: 20 prodromal PD-pRBD, 17 prodromal PD-npRBD	67.97 (5.70), 67.69 (5.97)	N.A.	N.A.	N.A.	TBSS	64	0/1000	-[PD-pRBD vs. PD-npRBD] widespread MD increases seen in prodromal PD-pRBD.
Wang et al. (2020)	43 PD-NC, 28 PD-MCI	60.19 (10.72), 63.93 (10.88)	24.00 (24.00), 24.00 (26.00) months	28.58 (10.68), 30.83 (13.83)	2.00 (1.00), 2.50 (1.00)	Graph theory, network-based analysis	26	0/1000	-[All PD vs. HC] reduced nodal efficiency in the hippocampus, parahippocampus, cingulate, temporal lobe, fusiform, and amygdala; the orbital nodal efficiency in PD in relation to overall cognitive function in PD - [PD-MCI vs. PD-NC] reduced nodal efficiency in the left olfactory cortex, left superior frontal gyrus, and medial orbital gyrus in PD-MCI
**Differential diagnosis of PD and atypical parkinsonism**									
Abos et al. (2019)	65 PD, 31 MSA	65.40 (10.00), 60.90 (8.40)	8.26 (6.02), 4.46 (2.75) years	16.59 (9.22), N.A.	[subject number, stage, 1/2/3/4/5] 10/32/22/1/0, 0/8/11/9/3	Network-based analysis, SVM for classification	30	0/1000	-[MSA vs. PD or HC] reduced number of fiber streamlines in connections between the bilateral striatum, ventral diencephalon, thalamus, and cerebellum in MSA - [MSA vs. PD] an accuracy of 0.78 for classification, with a sensitivity of 0.71, and a specificity of 0.86
Archer et al. (2019)	Multi-site data, 278 HC, 511 PD, 84 MSA, 129 PSP	Average age of all groups, 65.05 (9.65)	PD/MSA/PSP: 3.87 (3.84), 2.94 (2.77), 3.45 (3.16) years	PD/MSA/PSP: 30.15 (15.02), 51.13 (17.17), 40.65 (19.79)	N.A.	ROI analysis, SVM	[range] 15–64	0/1000 or 0/800	-Model only trained by DTI WM features achieving the best performance of AUC (PD vs. atyical parkinsonism: 0·955; MSA vs. PSP: 0·926)
Seki et al. (2019)	18 PSP, 16 MSA-P, 16 PD	67.10 (6.50), 63.90 (7.10), 65.20 (5.30)	2.30 (1.50), 1.90 (1.60), 3.20 (2.00) years	32.30 (9.00), 40.50 (7.20), 24.60 (6.90)	3.00 (0.375), 3.00 (1.00), 2.00 (1.00)	VBA, PCA, ROC analysis	20	0/1000	-MD and FA of the dentatorubrothalamic tract and CC were the most important components to achieve differential diagnosis. - [PSP vs. MSA or PD] an accuracy of 92% - [MSA-P vs. PD] an accuracy of 80%

ACC, anterior cingulate cortex; AD, axial diffusivity; ALIC, anterior limb of internal capsule; CC, corpus callosum; CR, corona radiata; CST, corticospinal tract; DLPFC, dorsal lateral prefrontal cortex; DTI, diffusion tensor imaging; EC, external capsule; FA, fractional anisotropy; FBA, fixel-based analysis; FC, fiber-bundle cross-section; FD, fiber density; FDC, fiber density and cross-section; FM, forceps mirror; H&Y, Hoehn-Yahr scale; HC, healthy control; IC, internal capsule; ICP, inferior cerebellar peduncle; IFG, inferior frontal gyrus; IFOF, inferior fronto-occipital fasciculus; ILF, inferior longitudinal fasciculus; L-PD, left onset PD; MCP, middle cerebellar peduncle; MD, mean diffusivity; MSA, multiple system atrophy; MSA-P, multiple system atrophy-parkinsonian type; NA, not available; NAWM, normal appearing white matter; NBM, nucleus Basalis of Meynert; PD-D, PD with dementia; PD-MCI, PD with mild cognitive impairment; PD-NC, PD with normal cognition; PD-npRBD, PD with no probable rapid eye movement sleep behavior disorder; PD-pRBD, PD with probable rapid eye movement sleep behavior disorder; PIGD-PD, postural instability and gait difficulty subtype of PD; PLIC, posterior limb of internal capsule; PPMI, Parkinson’s progression markers initiative; preSMA, pre-supplementary motor AREA; PSP, progressive supranuclear palsy; RBD, rapid eye movement sleep behavior disorder; RD, radial diffusivity; ROC, receiver operating characteristic; ROI, region of interest; R-PD, right onset PD; SCP, superior cerebellar peduncle; SD, standard deviation; SFOF, superior fronto-occipital fasciculus; SLF, superior longitudinal fasciculus; SST-CSD, single-shell 3-tissue constrained spherical deconvolution; STN, sub-thalamic nucleus; SVM, support vector machine; TBA, tract-based analysis; TBSS, tract-based spatial statistics; TD-PD, tremor dominant subtype of PD; UF, uncinate fasciculus; UPDRS-III, unified Parkinson’s disease Rating Scale part III; VBA, voxel-based analysis; WM, white matter; WMH, white matter hyperintensity.

## Recent advances in diffusion tensor imaging analytic methods

**Figure 1:**
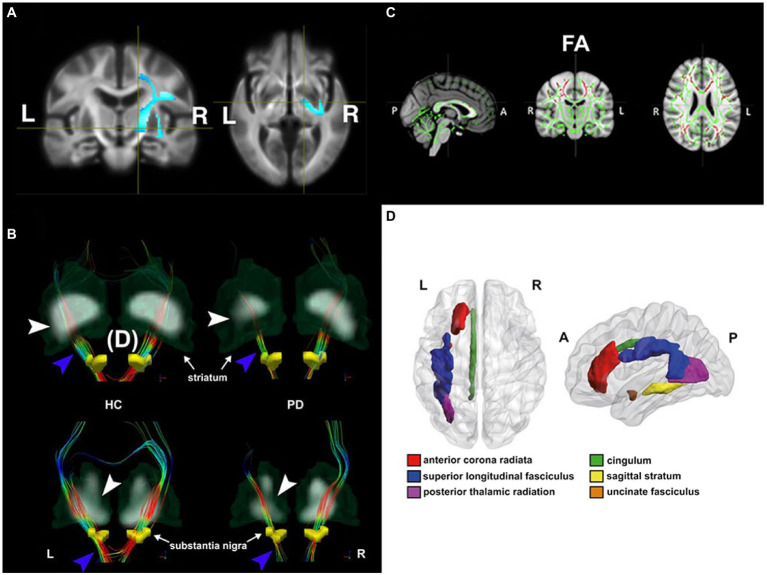
Diffusion changes in patients with Parkinson’s disease (PD) with different disease conditions shown by four different DTI analytic methods. **(A)** *De novo* PD stage: voxel-based analysis shows lower mean diffusivity (MD) in *de novo* PD patients relative to healthy controls (HC) in the voxels located at the right corticospinal tract, where the blue color-scale indicates the percentage of effect size. The darkest and lightest blue colors indicate 0 and 6% of the effect size, respectively. In addition, higher fiber cross-section in *de novo* PD patients was also found in the same brain region. Adapted from Xiao et al. (2021) with permission from John Wiley and Sons (34106502). **(B)** Early PD stage: tract-specific analysis using deterministic tractography delineates nigrostriatal fiber tracts (blue arrowhead) projecting from the substantia nigra (yellow regions of insterest [ROIs]) to the striatum in both HC (left) and PD (right) groups. The thinner nigrostriatal fiber tract in accordance with reduced striatal standardized uptake value ratio (white arrowhead) in the left side can be visualized in a PD patient. Color-encoded orientations: green for anterior–posterior, red for transverse, and blue for superior–inferior directions. Adapted from Shang et al. (2021) with permission from Springer Nature (34621005). **(C)** Mild to severe PD stages: patients showed decreased fractional anisotropy (FA, in red) in the tract bundles (e.g., genu, body, and splenium of corpus callosum, internal and external capsule, corona radiata, posterior thalamic radiation, sagittal stratum, cingulum and superior longitudinal fasciculus) detected by Tract-Based-Spatial-Statistics (TBSS) analysis. Slices from left to right are sagittal, coronal, and axial slices of the standard Montreal Neurological Institute (MNI) T1-weighted image template overlaid with the mean FA skeleton (in green). Adapted from Guimarães et al. (2018) with permission from Frontiers Research Foundation (30186216). **(D)** PD with depression: ROI analysis utilizes a between-group comparison of mean FA value in each ROI based on the predefined MRI atlas (Johns Hopkins University White Matter tractography atlas in this case). The figure illustrates the anatomical localization of FA change regions in MNI space in patients with depression. Adapted from Wu et al. (2018) with permission from BLACKWELL PUBLISHING (29125694).

Diffusion tensor imaging is sensitive to cortical microstructural changes in *de novo* PD patients, even with the absence of obvious atrophy observed on T1-weighted imaging (Tessa et al., 2008). Early DTI studies beyond past decades have drawn much attention to diffusion changes of the nigrostriatal pathway as an imaging hallmark in early PD, because this pathway links the substantia pars compacta (SNpc) to the striatum, where the dopaminergic depletion in the early PD stage will lead to the lack of motor control (Delong, 1990). More findings beyond the nigrostriatal circuit have been addressed with the recent advances in automated DTI analytic methods. These methodologies include the ROI analysis, voxel-based analysis (VBA), skeletonized approaches [i.e., Tract-based spatial statistics (TBSS; Smith et al., 2006], fixel-based analysis (FBA; Raffelt et al., 2015), and graph theory with network-based analysis (summarized in Figure 1).

In brief, ROI analysis is an automated method to extract the mean value of a DTI index from a predefined ROI, where ROIs can be obtained from automated segmentation based on different predefined anatomical MRI atlases. Johns Hopkins University (JHU) tractography atlas (Mori et al., 2008) has been widely used to utilize between-group comparison of a DTI index in a given fiber bundle ROI in PD studies (Wu et al., 2018; Juttukonda et al., 2019). VBA is a hypothesis-free approach to investigate microstructural properties in each voxel over the whole brain. Tract-Based Spatial Statistics (TBSS) is a popular automated approach to co-register individual DTI maps to the common DTI skeleton maps that allow statistical and objective group comparisons only on the voxels in the skeletonized white matter tracts (Smith et al., 2006). To date, TBSS is one of the most popular DTI analytic methods to investigate DTI changes due to PD with different neurodegenerative conditions (Wen et al., 2018; Ji et al., 2019; Chen et al., 2022). Unlike VBA investigating the overall diffusivity or anisotropy of a voxel, a fixel-based analysis is more likely to quantify the intravoxel white matter properties with the exclusion of other tissue compartments. To achieve this, each fixel is derived from the fiber orientation distributions obtained by the constrained spherical deconvolution (CSD) technique (Jeurissen et al., 2014). The graph-theoretical analysis is used to investigate differences in small-worldness, which is a measure to quantify global network efficiency and local connectivity strength within a large-scale structural topological organization that can be established by axonal interconnections (Bullmore and Sporns, 2009). This analysis could help understand network reorganizations in response to distinct dynamic pathophysiological changes in different PD subtypes or other parkinsonism disorders (Wen et al., 2018; Abos et al., 2019).

## Diffusion tensor imaging study in different Parkinson’s disease stages

DTI studies in patients with the prodromal stage of PD are still scarce. Rapid eye movement sleep behavior disorder (RBD) has been recently considered as a prodromal sign of PD. Growing evidence from neuroimaging studies indicates that pathophysiological changes substantially overlap between RBD and PD (Heller et al., 2017). Holtbernd et al. (2021) recently performed an ROI analysis to investigate the difference in DTI metrics of predefined brain atlas among PD patients, RBD patients, and healthy controls (HC). They identified the convergent pattern of axonal degeneration manifesting as lower FA in the corpus callosum and higher FA in the right corticospinal tract (CST) in both RBD and PD patients. The co-occurrence of FA changes in the opposite direction suggests that both the neurodegenerative and compensatory mechanisms are simultaneously proceeding, probably contributing to a continuous spectrum of PD development.

In the drug-naïve *de novo* Parkinson’s Progression Markers Initiative (PPMI) PD dataset, increased FA in the CST in PD patients was characterized by the fixel-based and VBA (Xiao et al., 2021). Notably, Xiao et al. (2021) also showed the applicability of clinical DTI data to the single-shell 3-tissue-constrained spherical deconvolution (SST-CSD) algorithm that is able to derive the extra diffusion metrics of fiber cross-section (FC) and fiber density (FD), accounting for gray matter (GM) and cerebrospinal fluid (CSF) compartments. The higher FC and FD were observed in the right posterior limb of the internal capsule (IC), cingulum, CST, and superior cerebellar peduncle (SCP). Increased FC in the motor pathways together with the above DTI findings, were interpreted as the consequence of neuroplastic reorganization against dopaminergic nigrostriatal degeneration during the *de novo* PD phase.

The recent studies in the middle to late PD stages moved to investigate microstructural changes in the brain regions beyond the nigrostriatal pathway, especially for those regions that appear structurally normal or unaffected by the pathophysiology of PD (Isaacs et al., 2019; Scamarcia et al., 2022). Scamarcia et al. (2022) performed both T2-weighted imaging and DTI to understand the longitudinal evolution of white matter damages, respectively, represented by increased white matter hyperintense (WMH) volume and altered DTI metrics in PD patients over time (one to four years). WMH volume gradually increased in patients over time. However, altered DTI metrics were only seen at baseline. Nonetheless, their baseline analysis revealed extensive axonal involvement in the normal-appearing white matter, with lower FA and higher diffusivity values in PD patients. These findings may suggest two different patterns of longitudinal evolution during the progressive PD course, with earlier development of microstructural changes conferring vulnerability to later WMH accumulation (Promjunyakul et al., 2018). In addition, Isaacs et al. (2019) aimed to determine whether the spatial registration to the standard stereotaxic space with the appropriate tractography atlas in the subthalamic nucleus (STN) can help identify more specific STN projections relevant to PD progression, and thereby improve the accuracy of DBS lead placement. They found significant FA reductions in the STN connections to the frontal areas, where the number of tract streamlines was comparable between PD and HC, suggesting higher sensitivity of DTI metrics than macrostructural measures. Lower FA in the STN connections was thought to affect preparatory motor control, task monitoring, and decision-making in PD.

## Diffusion tensor imaging changes in motor subtypes of Parkinson’s disease

### Postural instability and gait disorder dominant PD vs. tremor dominant PD

An early study performed manual ROI analysis and demonstrated lower FA and higher apparent diffusion coefficient values in the body of corpus callosum in PIGD-PD (Chan et al., 2014) relative to PD. Compared to the TD-PD subtype, PIGD-PD has been reported to have more severe gait and cognitive impairments, with more involvement in non-dopaminergic systems (Ren et al., 2020). As such, a recent DTI study comparing PIGD-PD vs. TD-PD shifted their attention to non-dopaminergic or non-motor brain regions. Nazmuddin et al. (2021) found a lower FA in the frontal NBM-WM tracts in PIGD-PD than TD-PD using VBA analysis on the predefined cholinergic white matter map innervated by nucleus basalis of Meynert white matter (NBM-WM) in the standard common MRI space. In addition, there were significant correlations among the severity of gait impairment, attentional performance, and NBM-WM DTI values in PD-PIGD. These findings indicate that PD-PIGD patients, even in the *de novo* stage, have already presented the impaired cholinergic white matter projections as a proxy of cholinergic denervation. Meanwhile, another DTI study performed the tract-specific analysis with probabilistic tractography to characterize white matter alterations between an executive-task-based functional network in both PIGD-PD and TD-PD with normal cognitive functions (Yang et al., 2021). They suggested that PIGD-PD patients exhibited poor performance on the FAS verbal fluency task relative to PD-T patients and more microstructural white matter impairments compared to PD-PIGD patients, in agreement with previous TBSS findings in both phenotypes at the *de novo* stage (Wen et al., 2018). Moreover, Schulz et al. (2018) depicted that degeneration of the nucleus basalis of Meynert measured by DTI predicts the onset of cognitive impairment; however, this was not confirmed in the PPMI cohort (Caspell-Garcia et al., 2017).

## Diffusion tensor imaging changes relevant to non-motor symptoms

### Normal cognition vs. mild cognitive impairment vs. dementia

Patients with PD can develop a spectrum of cognitive declines during disease progression. Compared with PD-NC, FA values in the corpus callosum, corona radiata, and cingulum substantially dropped in PD with dementia (PD-D) (Chondrogiorgi et al., 2019). TBSS revealed that all PD patients had lower cognitive performance assessed by the Parkinson’s disease-Cognitive Rating Scale in relation to FA/MD/AD in the prefrontal and limbic tracts as well as corpus callosum. Another DTI study further segmented the entire corpus callosum into several sub-regions and found that PD-D patients had increases in diffusivity metrics, not FA, in the most anterior callosal segment compared to HCs (Bledsoe et al., 2018), PD-NC, and PD with mild cognitive impairment (PD-MCI), suggesting the PD cognitive declines primarily caused by the disruption across interhemispheric callosal connections (Bledsoe et al., 2018; Chondrogiorgi et al., 2019). Minett et al. (2018) also applied TBSS to characterize widespread white matter impairments in both PD-NC and PD-MCI groups compared with HC beyond the corpus callosum and corona radiata, including the internal capsule, external capsule, and other association tracts. They also confirmed a longitudinal MD increase in the several frontal white matter tracts only in the PD-MCI group after 18 months, with no significant difference between PD and PD-MCI in any DTI metric at baseline. Based on their correlation results of baseline MD associated with executive/attention functions and longitudinal motor function declines, Minett et al. (2018) further suggested that MD can be serve as a predictor to monitor the progressive PD-related motor dysfunctions, and as a potential indicator to evaluate the effect of cognitive treatment on patients with PD-MCI or PD-D. Other approaches, such as network-based analysis (Wang et al., 2020) and hierarchical clustering analysis (Inguanzo et al., 2021), also demonstrated more severe deterioration in either network (e.g., less nodal efficiency) or DTI properties (e.g., reduced FA) in the frontal white matter network in PD-MCI patients compared with PD-NC.

## Differential diagnosis of Parkinson’s disease and atypical parkinsonism

It is challenging to differentiate PD, multiple system atrophy (MSA), and progressive supranuclear palsy (PSP), as these neurodegenerative diseases share some motor and non-motor features (Hughes et al., 2002). An increasing number of DTI studies have put considerable effort into differentiating MSA and/or PSP from PD over the past decade. In addition to classical statistical comparison, the recent DTI studies introduced a machine-learning approach with different DTI features to differentiate atypical parkinsonism from PD. Archer et al. (2019) recently proposed a novel machine-learning model based on a support vector machine (SVM) to classify patients with PD, MSA, or PSP. Their SVM models were, respectively, trained by the following combinations of features obtained from patients: diffusion MRI features only, Movement Disorder Society-Sponsored Revision of the Unified Parkinson’s Disease Rating Scale part-III (MDS UPDRS-III) only, diffusion MRI features + MDS UPDRS-III. In addition, they performed a free-water imaging method to reconstruct raw DTI data and produce novel diffusion MRI metrics [i.e., free-water (FW) and tissue FA (FA_T_)] as diffusion MRI features that can correct the partial volume effect of corticospinal fluid on FA value (Pasternak et al., 2009). Several PD studies have used free water imaging to consistently show increased FW within the posterior substantia nigra in PD (Planetta et al., 2016). Critically, their SVM model can achieve the best performance for classifying PD, MSA, and PSP when only using FW and FA_T_ features. Another study (Abos et al., 2019) also performed SVM models, respectively, trained by DTI metrics, subcortical volume, or the number of fiber streamlines to distinguish patients with MSA from patients with PD. Finally, Seki et al. (2019) adopted a multimodal MRI approach to extract different quantitative MRI features based on VBA results. The following principal component analysis was used to reduce the high dimensionality of MRI features, and stepwise receiver-operating characteristic curve analysis was used to distinguish patients with PSP, Parkinson type of MSA (MSA-P), and PD. Notably, principal component analysis also highlighted DTI metrics of a few critical clusters to differentiate the three patient cohorts. These clusters were mainly distributed at the thalamus, dentatorubrothalamic tract, corpus callosum, and middle cerebellar peduncle. These findings were consistent with previous MRI findings (Nicoletti et al., 2006; Ito et al., 2008), where the axonal degeneration of dentatorubrothalamic tract comprises of demyelinated fibers, tau pathology, and microgliosis seen in autopsy PSP subjects (Ishizawa and Dickson, 2001).

## Deep brain stimulation therapy for Parkinson’s disease

DBS is a conventional therapy for PD when treatment with dopaminergic medication is inadequate. However, the variability of DBS therapy sometimes proposes a need for a more detailed characterization of the targeted networks and visualization of white matter pathways that drives the clinical outcome (Henderson, 2012; Calabrese, 2016; Gonzalez-Escamilla et al., 2022). DT has been proposed to be the only non-invasive method of visualizing neural structural connectivity that could guide DBS targeting in neurological diseases, including PD (Calabrese, 2016). DT can define specific axonal trajectories as the basis of pathway-activation models to provide better anatomical/electrical volume conductor estimations to evaluate the strength and coverage of electric field covering DBS targets (Gunalan et al., 2018). As such, DT helped strategize the best direct or indirect DBS target by delineating and segmenting STN-cortical (Chen et al., 2018; Gunalan et al., 2018), pallido-cortical (Middlebrooks et al., 2018), pedunculopontine (Raghu et al., 2021), and cortico-cerebellar pathways (Strotzer et al., 2019; Coenen et al., 2020) to mitigate PD-related motor symptoms and minimize unwanted side effects, such as depression and impaired cognition (Combs et al., 2015; Irmen et al., 2020).

In another recent study, Gonzalez-Escamilla et al. (2022), using DTI and probabilistic tractography, presented an approach to integrate postoperative MRI, showing the DBS contact locations, with WM pathways connecting the contact locations to essential parts of motor network in individual PD patients. Specifically, the authors showed that the postoperative outcome of STN-DBS is strongly associated with active stimulation of contacts connected to the primary cortex and supplementary motor area and that can be individually defined. In addition, both DTI profile and network-based connectivity have served as a preoperative predictor for postoperative outcome (Koirala et al., 2018; Gonzalez-Escamilla et al., 2022) or a targeted indicator to trace the therapeutic effect (Strotzer et al., 2019; Huang et al., 2022).

## Conclusion

The above studies summarized in the Table 1 indicate that DTI, measuring changes in white matter microstructure, specifically axonal changes, might be more sensitive in the early PD stage than other imaging techniques. DTI can characterize structural changes related to PD as the disease progresses over time so it can be a promising biomarker for monitoring PD progression. It also has the potential for making a more sensitive and specific differential diagnosis between PD and atypical parkinsonism, such as MSA and PSP (de Oliveira and Pereira, 2017). DTI also sheds light on the underlying structural network affected in different subtypes of PD (PD-T vs. PD-PIGD), which can help future interventional studies offering optimized treatment to various subtypes (e.g., treating PD-PIGD patients, in the *de novo* stage with the impaired cholinergic white matter projections as a proxy of cholinergic denervation with the novel DBS techniques being developed to increase cholinergic modulation).

Moreover, identifying the network topology and connectivity using DTI and probabilistic tractography before traditional DBS surgery directly influences PD patients’ response to DBS and may serve as significant predictors of the DBS clinical outcome (Koirala et al., 2018). Finally, structural changes, detected by DTI, are not only linked to motor but also cognitive symptoms. Therefore, DTI can potentially characterize the structural network involved in PD cognitive impairment leading to dementia and differentiate PD patients with mild cognitive impairment from those with normal cognition. Moreover, DTI information linked with machine learning approaches will add predictive power to this technique to act as a biomarker for early detection of those PD patients at risk of cognitive decline.

## Author contributions

Y-CS and LM-K contributed to writing the manuscript. W-YT contributed to editing the manuscript. All authors contributed to the article and approved the submitted version.

## Conflict of interest

W-YT was employed by AcroViz Inc. The remaining authors declare that the research was conducted in the absence of any commercial or financial relationships that could be construed as a potential conflict of interest.

## Publisher’s note

All claims expressed in this article are solely those of the authors and do not necessarily represent those of their affiliated organizations, or those of the publisher, the editors and the reviewers. Any product that may be evaluated in this article, or claim that may be made by its manufacturer, is not guaranteed or endorsed by the publisher.
